# Injuries Common to the Brazilian Jiu-Jitsu Practitioner

**DOI:** 10.7759/cureus.37502

**Published:** 2023-04-12

**Authors:** James J Hunker, Sandip P Tarpada, Joseph Khoury, Abraham Goch, Mani Kahn

**Affiliations:** 1 Surgery, New York Presbyterian - Weill Cornell Medical Center, New York City, USA; 2 Department of Orthopaedic Surgery, Montefiore Medical Center, Wakefield Campus, Bronx, USA; 3 Medicine, Albert Einstein College of Medicine, New York City, USA; 4 Department of Orthopaedic Surgery, Inova Medical Group, Church Falls, USA

**Keywords:** injuries, sports medicine, orthopaedics, martial arts, brazilian jiu-jitsu

## Abstract

Background: Brazilian jiu-jitsu (BJJ) is a martial art that focuses on ground combat, emphasizing skill over strength and submission over striking. The purpose of this study is to evaluate the nature of injuries affecting practitioners of BJJ in the settings of competition, training, and conditioning.

Methods: An online survey was created to collect demographic and injury-specific information. This survey was distributed to the 234 schools in the United States registered with the International Brazilian Jiu-Jitsu Federation (IBJJF). The survey was also distributed to local BJJ schools and at local tournaments in the Greater New York City area. Data from a total of N=56 participants were recorded for this survey.

Results: The majority of participants were male (n=44, 78.6%) and amateur competitors (n=29, 51.8%) with an average duration of BJJ training of 6.9 ± 5.9 years. The majority of participants (82.1%) train at least six hours per week and compete in an average of 4.6 ± 2.5 competitions per year. The most common injuries were to the finger/hand (78.6%) and knee (61.5%). The most commonly reported fracture was of the hand/fingers (n=6). Of the 156 total injuries reported, 133 (85.3%) occurred during practice or training rather than in competition and 76 (48.7%) required medical attention. Few injuries required surgical intervention.

Conclusions: This study provides novel information regarding injury characteristics of BJJ practitioners with respect to the level of training and use of protective equipment that can guide expectations and management for this unique group of athletes. Amateur BJJ practitioners are the most commonly injured, and largely experience injuries of the upper extremities during training or conditioning rather than during competition.

## Introduction

Brazilian jiu-jitsu (BJJ) is a martial art that focuses on ground combat, emphasizing grappling skill over strength and submission over striking. First developed in the early 19th century, it rapidly gained popularity in America with the creation of the Ultimate Fighting Championship (UFC) in 1993 [1]. BJJ continues to be a popular and growing sport in the United States today [2,3]. Practitioners of BJJ typically wear a belt that denotes their rank and skill. The belt ranks for adults (age 16 and older) in increasing order of rank, are White, Blue, Purple, Brown, Black, Red with Black, Red with White, and Red. Graduation for belts is dependent on duration, skill, and a minimum age. The youngest age at which a black belt can be earned is 19. Belts, in addition to weight class and age, form competition groups by which tournaments are organized [4]. Given the substantial variability in age and ranking of BJJ practitioners, a vast spectrum of orthopaedic injuries results in this population. Unsurprisingly, this poses a unique clinical challenge for orthopaedic providers treating these injuries [3].

While mixed martial arts (MMA) injuries have been the focus of many extensive studies, BJJ has not received nearly as much attention [5,6]. To our knowledge, there is scant research on injuries that occur during BJJ competition, with only three studies including injuries that occur during training, and no research regarding injuries sustained during BJJ-specific conditioning [3,7-10]. The purpose of this study is to enhance the understanding of injuries that occur in BJJ by analyzing the prevalence of injury with regard to the specific environment and skill level. In determining the characteristics of the most commonly observed injuries in BJJ in the settings of competition, training, and conditioning, this study aims to improve the care of the growing population of BJJ practitioners.

## Materials and methods

This study was reviewed and approved by the institutional review board (IRB) of Montefiore Medical Center. An online survey was created to collect demographic and injury-specific information (Table 1). This survey was distributed to the 234 BJJ schools in the United States registered with the International Brazilian Jiu-Jitsu Federation (IBJJF). The survey was also distributed to local BJJ schools and at local tournaments in the Greater New York City area.

**Table 1:** Brazilian jiu-jitsu (BJJ) injuries survey distributed to athletes in plain text format

This questionnaire is part of a medical research project to better understand injuries common to practitioners of Brazilian Jiu-Jitsu. All participants must be at least 18 years old. All information provided will be anonymous. In order to maintain anonymity, upon submission of this questionnaire there will be a link to access a separate form to enter the raffle. Your responses to the survey do NOT affect your chances to win.	
What is your age?	
What is your sex?	
What is your belt rank: white, blue, purple, brown, black, none, other?	
How many years of training have you had?	
How many hours per week do you train/practice?	
What protective equipment do you use?	
What is your current competitor status: non-competitor, amateur, professional?	
How many BJJ competitions per year do you participate in?	
What is your weight class?	
Have you sustained injuries from BJJ that required either medical attention or an interruption/break from normal BJJ practice?	
Have you sustained injuries to your hand/fingers? If so, please describe.	
Did this injury occur in competition, practice/sparring, or conditioning? Did you seek medical attention or require surgery?	
Have you sustained injuries to your wrist/forearm? If so, please describe.	
Did this injury occur in competition, practice/sparring, or conditioning? Did you seek medical attention or require surgery?	
Have you sustained injuries to your elbow/upper arm? If so, please describe.	
Did this injury occur in competition, practice/sparring, or conditioning? Did you seek medical attention or require surgery?	
Have you sustained injuries to your shoulder? If so, please describe.	
Did this injury occur in competition, practice/sparring, or conditioning? Did you seek medical attention or require surgery	
Have you sustained injuries to your head/face? If so, please describe.	
Did this injury occur in competition, practice/sparring, or conditioning? Did you seek medical attention or require surgery?	
Have you sustained injuries to your neck? If so, please describe.	
Did this injury occur in competition, practice/sparring, or conditioning? Did you seek medical attention or require surgery?	
Have you sustained injuries to your upper back? If so, please describe.	
Did this injury occur in competition, practice/sparring, or conditioning? Did you seek medical attention or require surgery?	
Have you sustained injuries to your lower back? If so, please describe.	
Did this injury occur in competition, practice/sparring, or conditioning? Did you seek medical attention or require surgery?	
Have you sustained injuries to your chest/ribs? If so, please describe.	
Did this injury occur in competition, practice/sparring, or conditioning? Did you seek medical attention or require surgery?	
Have you sustained injuries to your abdomen? If so, please describe.	
Did this injury occur in competition, practice/sparring, or conditioning? Did you seek medical attention or require surgery?	
Have you sustained injuries to your hip/groin/pelvis? If so, please describe.	
Did this injury occur in competition, practice/sparring, or conditioning? Did you seek medical attention or require surgery?	
Have you sustained injuries to your thigh/gluteus? If so, please describe.	
Did this injury occur in competition, practice/sparring, or conditioning? Did you seek medical attention or require surgery?	
Have you sustained injuries to your knee? If so, please describe.	
Did this injury occur in competition, practice/sparring, or conditioning? Did you seek medical attention or require surgery?	
Have you sustained injuries to your lower leg/ankle? If so, please describe.	
Did this injury occur in competition, practice/sparring, or conditioning? Did you seek medical attention or require surgery?	
Have you sustained injuries to your foot/toes? If so, please describe.	
Did this injury occur in competition, practice/sparring, or conditioning? Did you seek medical attention or require surgery?	

Athletes over the age of 18 were included regardless of a past history of injury. The 56 survey-responding athletes were asked to provide demographic data as well as information regarding protective equipment and injuries sustained during BJJ competitions, training, and conditioning. Competitor status is divided according to the level of the competitions the athletes participate in. A non-competitor is someone who trains but does not compete, an amateur is someone who competes on their own time and/or pays for it, and a professional is someone who competes in BJJ full-time. Participants were additionally asked to include and describe any and all injuries that caused an interruption or modification of normal training or injuries for which they sought medical care. Within the survey, injuries were separated by “body part” as follows: hand/fingers, wrist/forearm, elbow/upper arm, shoulder, head/face, neck, upper back, lower back, chest/ribs, abdomen, hip/groin/pelvis, thigh/gluteus, knee, lower leg/ankle, foot/toes. As an incentive for participation, athletes who completed the survey were able to enter a raffle to win one of four $50 gift cards. To protect the health information and privacy of participants, the raffle entry was kept separate from the research survey. The statistical data was analyzed via XLMiner using student’s t-test, and categorical variables were analyzed using Fisher’s exact test.

## Results

Participant characteristics

The questionnaire responses were recorded for 56 participants, of which 41 (73.2%) reported sustaining an injury with a total number of body parts injured per BJJ athlete of 2.9 ± 2.4. The majority of participants were male (n=44, 78.6%) and amateur competitors (n=29, 51.8%). Full demographic data is provided (Table 2). The average duration of BJJ training was 6.9 ± 5.9 years. The most common belt ranks were blue (26.8%) and black (23.2%) and the majority of participants (82.1%) train at least six hours per week. Nearly half of participants endorse using protective equipment (44.6%) and the most commonly used protective equipment was a mouth guard (n=21, 38.2%) (Table 3).

**Table 2:** Demographics of participants per competitor group

Demographics	Non-competitor (N=16)	Amateur competitor (N=29)	Professional competitor (N=11)	Total (N=56)
Age (years)				
18-21	0	1 (3.4%)	1 (9.1%)	2 (3.6%)
22-25	0	5 (17.2%)	6 (54.5%)	11 (19.6%)
26-29	1 (6.3%)	6 (20.7%)	1 (9.1%)	8 (14.3%)
30-33	1 (6.3%)	4 (13.8%)	2 (18.2%)	7 (12.5%)
34-37	0	8 (27.6%)	0	8 (14.3%)
38-41	5 (31.3%)	2 (6.9%)	0	7 (12.5%)
42-45	2 (12.5%)	1 (3.4%)	0	3 (5.4%)
45+	7 (43.8%)	2 (6.9%)	1 (9.1%)	10 (17.9%)
Gender				
Female	2 (12.5%)	7 (24.1%)	3 (27.3%)	12 (21.4%)
Male	14 (87.5%)	22 (75.9%)	8 (72.2%)	44 (78.6%)
Belt rank				
White	1 (6.3%)	7 (24.1%)	0	8 (14.3%)
Blue	1 (6.3%)	11 (37.9%)	3 (27.3%)	15 (26.8%)
Purple	4 (25%)	4 (13.8%)	2 (18.2%)	10 (17.9%)
Brown	1 (6.3%)	4 (13.8%)	5 (45.5%)	10 (17.9%)
Black	9 (56.3%)	3 (10.3%)	1 (9.1%)	13 (23.2%)
Weight Class				
Feather/Light	3 (18.8%)	4 (13.8%)	2 (18.2%)	9 (16.1%)
Feather	5 (31.3%)	6 (20.7%)	2 (18.2%)	13 (23.2%)
Light	2 (12.5%)	4 (13.8%)	2 (18.2%)	8 (14.3%)
Middle	3 (18.8%)	3 (10.3%)	0	6 (10.7%)
Heavy	2 (12.5%)	1 (3.4%)	2 (18.2%)	5 (8.9%)
Medium heavy	1 (6.3%)	8 (27.6%)	2 (18.2%)	11 (19.6%)
Super Heavy	0	3 (10.3%)	1 (9.1%)	4 (7.1%)
Years Training	12 ± 7.7	4.4 ± 3.4	6.4± 3.6	6.9 ± 5.9
Hours Training				
0-2	1 (6.3%)	0	0	1 (1.8%)
3-5	3 (18.8%)	5 (17.2%)	1 (9.1%)	9 (16.1%)
6-8	4 (25%)	16 (55.2%)	1 (9.1%)	21 (37.5%)
9-11	2 (12.5%)	5 (17.2%)	4 (36.4%)	11 (19.6%)
12+	6 (37.5%)	3 (10.3%)	5 (45.5%)	14 (25%)
Protective Equipment				
No	9 (56.3%)	14 (48.3%)	8 (72.7%)	31 (55.4%)
Yes	7 (43.8%)	15 (51.7%)	3 (27.3%)	25 (44.6%)
Injury				
No	2 (12.5%)	9 (31%)	4 (36.4%)	15 (26.8%)
Yes	14 (87.5%)	20 (69%)	7 (63.6%)	41 (73.2%)
Competitions per year		3.9 ± 2.2	6.8 ± 1.9	4.6 ± 2.5

**Table 3:** Usage of protective equipment per competitor group

	Non-competitor (N=16)	Amateur competitor (N=29)	Professional competitor (N=11)	Total (N=56)
Mouth Guard				
No	10 (62.5%)	16 (55.2%)	8 (80%)	34 (61.8%)
Yes	6 (37.5%)	13 (44.8%)	2 (20%)	21 (38.2%)
Knee Pads				
No	15 (93.8%)	26 (89.7%)	10 (100%)	51 (92.7%)
Yes	1 (6.3%)	3 (10.3%)	0	4 (7.3%)
Finger Tape				
No	16 (100%)	28 (96.6%)	9 (90%)	53 (96.4%)
Yes	0	1 (3.4%)	1 (10%)	2 (3.6%)
Groin Protector				
No	16 (100%)	27 (93.1%)	10 (100%)	53 (96.4%)
Yes	0	2 (6.9%)	0	2 (3.6%)

Injuries

**Figure 1:**
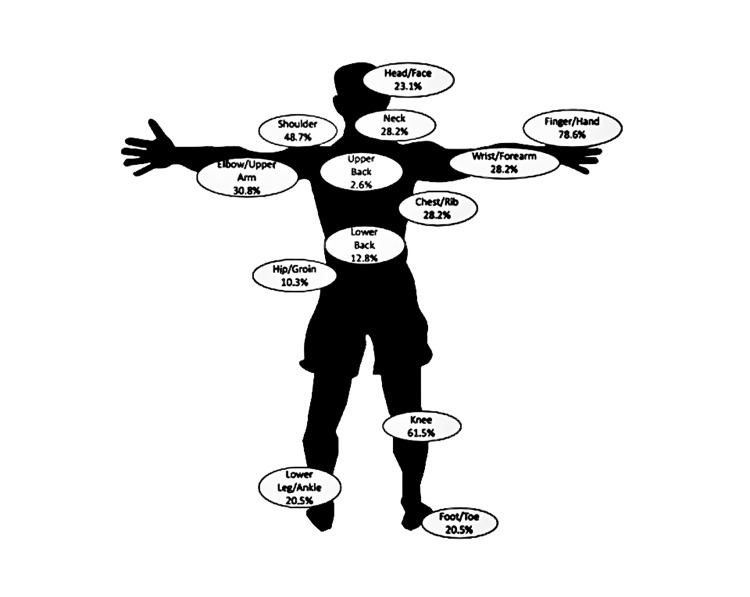
Total percentage of injuries per body part This figure is the author's own creation.

There was a total of 156 injuries reported; 116 (74.4%) were to the upper body and 40 (25.6%) were to the lower body. Of the 156 total injuries, 133 (85.3%) occurred during practice or training, rather than in competition. The most common injuries were to finger/hand (78.6%) and knee (61.5%); the least common injuries were to the abdomen (0) and thigh/gluteus (0) (Table 4, Figure 1). Of the 156 injuries reported, 76 received medical attention (48.7%). Fifteen of the 56 athletes (27%) had undergone surgery for a BJJ injury.

**Table 4:** Summary of injuries by body part per competitor group

Injury	Non-competitor	Amateur competitor	Professional competitor	Total (156)
Upper Body Injuries				116
Finger/Hand	11 (73.3%)	15 (75%)	7 (100%)	33 (78.6%)
Wrist/Forearm	3 (25%)	6 (30%)	2 (28.6%)	11 (28.2%)
Elbow/Upper Arm	4 (33.3%)	4 (20%)	4 (57.1%)	12 (30.8%)
Shoulder	6 (50%)	7 (35%)	6 (85.7%)	19 (48.7%)
Head/Face	3 (25%)	5 (25%)	1 (14.3%)	9 (23.1%)
Neck	5 (41.7%)	5 (25%)	1 (14.3%)	11 (28.2%)
Upper Back	1 (8.3%)	0	0	1 (2.6%)
Lower Back	2 (16.7%)	2 (10%)	1 (14.3%)	5 (12.8%)
Chest/Rib	5 (41.7%)	2 (10%)	4 (57.1%)	11 (28.2%)
Abdomen	0	0	0	0
Hip/Groin	1 (8.3%)	1 (5%)	2 (28.6%)	4 (10.3%)
Thigh/Gluteus	0	0	0	0
Lower Body Injuries				40
Knee	8 (66.7%)	11 (55%)	5 (71.4%)	24 (61.5%)
Lower Leg/Ankle	3 (25%)	4 (20%)	1 (14.3%)	8 (20.5%)
Foot/Toe	2 (16.7%)	4 (20%)	2 (28.6%)	8 (20.5%)
Number of body parts injured per Brazilian Jiu-Jitsu (BJJ) athlete	3.9 ± 2.3	2.3 ± 2.1	3.2 ± 3.1	2.9 ± 2.4
Number of surgeries per BJJ athlete	0.4 ± 0.6	0.3 ± 0.5	0.1 ± 0.3	0.3 ± 0.5

Injuries to the Upper Extremity

There was a total of 75 upper extremity injuries in our cohort, occurring most commonly to the finger and hand (n=33; Table 5). Of the injuries to the hand, finger injuries were the most prevalent at 63.3%. Furthermore, the most common type of finger and hand injury was a sprain/strain which was reported 24.6% of the time. There were no significant differences in rates of finger/hand injury (p = 0.496), or rates of wrist/forearm injury (p = 0.897) among the three competitor types (non-competitor, amateur competitor, and professional competitor). Of injuries to the fingers or hand, 87.5% occurred in practice or training rather than competition and half required medical attention. Further, 78.1% (25 of 32) required a modification in training although the majority (65.6%) did not require a break from training. The fingers or hand were the body part with the most reported fractures (n=6). Of injuries to the wrist/forearm, 90.9% occurred in practice or training and 45.5% required medical attention, 81.8% modified training, and 45.5% took a break from training. For the elbow/upper arm, half required medical attention, 83.3% had to modify training, and half took a break from training (Table 6). Relative to others, there was a higher occurrence of these injuries in competition (relative risk = 3.5). Shoulder injuries occurred almost exclusively in practice/training (94.7%) but most did not seek medical attention (57.9%). 78.9% modified training and 73.7% took a break from training.

**Table 5:** Finger or hand injury characteristics

	Non-competitor (N=14)	Amateur competitor (N=20)	Professional competitor (N=7)	Total (N=41)
Finger/Hand	11 (73.3%)	15 (75%)	7 (100%)	33 (78.6%)
Finger or Hand Injury				
Knuckle	1 (10%)	4 (28.6%)	2 (33.3%)	7 (23.3%)
Palm	0	0	1 (16.7%)	1 (3.3%)
Finger	8 (80%)	8 (57.1%)	3 (50%)	19 (63.3%)
Wrist	1 (10%)	0	0	1 (3.3%)
Joints	0	2 (14.3%)	0	2 (6.7%)
Type of Injury	N/A	N/A	N/A	
Dislocation				10 (15.4%)
Fracture				6 (9.2%)
Jam				9 (13.8%)
Sprain/strain				16 (24.6%)
Ligament/tendon injury				14 (21.5%)
Hyperextension				10 (15.4%)
Practice/Competition				
Practice	10 (100%)	12 (80%)	6 (85.7%)	28 (87.5%)
Competition	0	3 (20%)	1 (14.3%)	4 (12.5%)
Medical Attention				
No	6 (60%)	8 (53.3%)	2 (28.6%)	16 (50%)
Yes	4 (40%)	7 (46.7%)	5 (71.4%)	16 (50%)
Require Surgery				
No	10 (100%)	15 (100%)	6 (85.7%)	31 (96.9%)
Yes	0	0	1 (14.3%)	1 (3.1%)
Modified Training				
No	3 (30%)	3 (20%)	1 (14.3%)	7 (21.9%)
Yes	7 (70%)	12 (80%)	6 (85.7%)	25 (78.1%)
Modified Training	N/A	N/A	N/A	
Use tape				9 (31%)
Adjust grip				5 (17.2%)
Take a break				3 (10.3%)
Lighten up on move(s)				2 (6.9%)
Avoid move(s)				6 (20.7%)
Don’t use hand				3 (10.3%)
Use a brace				1 (3.4%)
Break in Training				
No	5 (50%)	12 (80%)	4 (57.1%)	21 (65.6%)
Yes	5 (50%)	3 (20%)	3 (42.9%)	11 (34.4%)
Length of Break				
1-14 days	4 (100%)	2 (66.7%)	0	6 (60%)
14+ days	0	1 (33.3%)	3 (100%)	4 (40%)

**Table 6:** Wrist or forearm injury characteristics

	Non-competitor (N=14)	Amateur competitor (N=20)	Professional competitor (N=7)	Total (N=41)
Wrist/Forearm Injury	3 (25%)	6 (30%)	2 (28.6%)	11 (28.2%)
Type of Injury	N/A	N/A	N/A	
Strain				8 (57.1%)
Ligament/tendon injury				2 (14.3%)
Hyperextension				3 (21.4%)
Other				1 (7.1%)
Practice/Competition				
Practice	3 (100%)	5 (83.3%)	2 (100%)	10 (90.9%)
Competition	0	1 (16.7%)	0	1 (9.1%)
Medical Attention				
No	2 (66.7%)	3 (50%)	1 (50%)	6 (54.5%)
Yes	1 (33.3%)	3 (50%)	1 (50%)	5 (45.5%)
Require Surgery				
No	3 (100%)	4 (66.7%)	2 (100%)	9 (81.8%)
Yes	0	2 (33.3%)	0	2 (18.2%)
Modified Training				
No	1 (33.3%)	1 (16.7%)	0	2 (18.2%)
Yes	2 (66.7%)	5 (83.3%)	2 (100%)	9 (81.8%)
Break in Training				
No	2 (66.7%)	4 (66.7%)	0	6 (54.5%)
Yes	1 (33.3%)	2 (33.3%)	2 (100%)	5 (45.5%)
Length of Break				
1-14 days	0	1 (50%)	1 (100%)	2 (50%)
14+ days	1 (100%)	1 (50%)	0	2 (50%)

Injuries to the Head and Trunk

There were 37 injuries reported to the head and trunk, most commonly affecting the neck (n=11) and chest/ribs (n=11). For injuries to the head and face, 77.8% occurred in practice rather than competition. Nearly half of these injuries required medical attention and two-thirds took a break from training but none required surgery. Injuries to the neck were reported only in practice and training rather than competition. The most common types of injuries to the neck were sprain/strain (41.2%) and disc injury (23.5%), 63.6% required medical attention, and 63.6% modified training.

Importantly, 90.9% of these patients took a hiatus from training and 70% of those athletes required longer than two weeks to recover before resuming training. Injuries to the upper back and abdomen were uncommon. There were few reported injuries to the lower back, all of which occurred in practice. Medical attention was sought for 20% and no surgeries were reported. These injuries did appear to require a long recovery where 80% took a break from training and more than two-thirds took a break longer than two weeks. Reported chest and rib injuries all required a modification in training and were the second most common fractures reported by body part (n=5). For the chest and rib injuries, 63.6% required a break from training of at least two weeks.

Lower Extremity Injuries

There was a total of 44 injuries reported to the lower extremity with the knee being the most common of these (n=24; Table 7). There were no reported injuries to the thigh or gluteus. Injuries to the hip and groin were reported only in practice (n=4; Table 4). Of these 44 lower extremity injuries, 75% had to modify their training. There were 24 knee injuries reported, most commonly to the meniscus, and 91.7% of reported knee injuries occurred in practice rather than competition. Two-thirds of these injuries required medical attention and knee injuries were most likely to require surgery. There were no significant differences in rates of knee injury among the three competitor types (p = 0.835). For reported injuries to the lower leg and ankle, 62.5% occurred in practice. Injuries to the foot and toes were relatively common, including fractures and 87.5% of these injuries occurred in practice. Few required medical attention, commonly self-treated with a respite from activity and buddy-taping.

**Table 7:** Knee injury characteristics

	Non-competitor (N=14)	Amateur competitor (N=20)	Professional competitor (N=7)	Total (N=41)
Knee Injury	8 (66.7%)	11 (55%)	5 (71.4%)	24 (61.5%)
Practice/Competition				
Practice	8 (100%)	9 (81.8%)	5 (100%)	22 (91.7%)
Competition	0	2 (18.2%)	0	2 (8.3%)
Medical Attention				
No	2 (25%)	3 (27.3%)	3 (60%)	8 (33.3%)
Yes	6 (75%)	8 (72.7%)	2 (40%)	16 (66.7%)
Require Surgery				
No	4 (50%)	7 (63.6%)	5 (100%)	16 (66.7%)
Yes	4 (50%)	4 (36.4%)	0	8 (33.3%)
Modified Training				
No	1 (12.5%)	1 (9.1%)	0	2 (8.3%)
Yes	7 (87.5%)	10 (90.9%)	5 (100%)	22 (91.7%)
Break in Training				
No	0	3 (27.3%)	1 (20%)	4 (16.7%)
Yes	8 (100%)	8 (72.7%)	4 (80%)	20 (83.3%)
Length of Break				
1-14 days	1 (12.5%)	2 (18.2%)	3 (60%)	6 (25%)
14+ days	7 (87.5%)	6 (54.5%)	1 (20%)	14 (58.3%)

Use of safety equipment

Less experienced and junior belt athletes were more likely to use protective equipment. Sixty-three percent of white belts report using a mouth guard compared to only 15% of black belts.

## Discussion

Brazilian jiu-jitsu has rapidly become a widely popular sport, with an estimated following of nearly 3 million practitioners worldwide [1,3]. Despite this, there exists a paucity of literature elucidating the injury profile of BJJ practitioners during either training or competition. Recently Petrisor et al. conducted a descriptive epidemiological, survey-based study on 70 BJJ athletes belonging to a single club in Ontario, Canada [3]. They found that 91% of BJJ practitioners suffered at least one injury during training, and 60% of athletes were injured during competition. The most commonly affected locations of injury included the hand/fingers (37% of injuries) and nearly two-thirds of those injured required some form of formal medical intervention. They additionally found that a history of requiring surgery for an injured extremity resulted in a 6.5 times higher risk of failing to return to sport. They conclude that the high prevalence of injury, and subsequent care received, among BJJ practitioners both play a significant psychological and physical role in the participant’s future function within the sport.

Our findings, in part, corroborate those of Petrisor et al. (2019). The vast majority (85.3%) of injuries among our survey population occur during training or conditioning rather than in competition. Furthermore, among practitioners incurring an injury, we find that half subsequently sought out formal medical attention. This figure appears to be consistent with the literature available for other forms of martial arts such as karate, judo, taekwondo and MMA [3,11,12]. While the survey population of Petrisor et al. includes equal numbers of recreational athletes and competitive athletes, we found there to be substantially fewer competition-level athletes in our population, likely a result of our inclusion of many local BJJ schools compared to the single facility scrutinized within their study.

Unsurprisingly, experienced athletes were found to be more likely to have a history of prior injury. The use of mouth guards, knee pads, finger tape, and groin protectors during training and competition have a demonstrated track record of preventing injury within a multitude of combat sports [13-15]. Despite this, we found that more experienced athletes used protective equipment less frequently than those with less experience. The most common injuries reported amongst experienced athletes were finger/hand injuries (7). Finger tape appears to be almost unanimously used among all levels of training and competition. Interestingly however, the use of a circumferential pulley tape has been found to be biomechanically disadvantageous and may even lead to an increased risk of injury in the uninjured finger [16-18]. Our findings lead us to conclude that further research specifically addressing the role of preventative finger taping within BJJ is needed.

We found the most common injuries among BJJ practitioners to involve the upper extremity, including hands and fingers, and the knee. Injuries to the fingers and hand were most common regardless of belt rank or competitor status and also incurred the most fractures suggesting that the fingers and hand are particularly vulnerable to BJJ injury. In a 2014 study by Scoggin et al., use of the “arm bar” maneuver during competition was the most common mechanism of upper extremity injury [9]. Similarly, McDonald et al. (2019) found hand and finger injuries to occur most frequently during competition, and to be the injury least likely to be reported to medical officials after competition. Our data uniquely shows that injury type and location vary in incidence by setting. For example, of the 11 reported neck injuries, all occurred in practice whereas, of the 12 reported elbow/upper arm injuries, only 58.3% occurred in practice with the remaining occurring in competition.

From the physician’s perspective, the neck represents a particularly vulnerable area of injury. Our results supported this concern as reported neck injuries appeared particularly severe with 90.9% of injuries to the neck requiring a break in training and 70% of those disruptions in training lasted longer than two weeks.

Limitations

This study is primarily limited by its low sample size. Although 234 International BJJ registered schools were surveyed, along with other local New York City BJJ tournaments and schools, the fleeting nature of available tournaments certainly contributed to an overall low sample number. For this reason, our study may not be powered to make decisive claims. However, our descriptive analysis of the given data adds valuable information to the orthopaedic literature surrounding common BJJ injuries, particularly given the paucity of currently available literature. Additionally, we acknowledge the elements of recall bias, selection bias, and self-reporting bias inherent within survey studies of this nature. The potential for recall bias was mitigated by using objective measures of injury. The potential for selection bias was mitigated by sampling BJJ participants from 234 different schools around the United States, as well as from local NYC schools/tournaments. The potential for self-reporting bias was mitigated by ensuring that the survey results remained anonymous. Additional shortcomings of our study include the length of the survey, which may have been cumbersome for some participants, and the availability only in English. Information regarding injury type and setting of injury within BJJ is very limited, and the inclusion of our study may pave the road for further inquiry, including mechanisms of injury prevention.

## Conclusions

With the growing sport of MMA, more athletes are likely to participate in multi-disciplinary training, of which BJJ is a common component. Here we demonstrate that injury among BJJ practitioners occurs more frequently during training, rather than competition. The most common injuries found among BJJ practitioners occur to the hands/fingers and the knee. Furthermore, senior-ranking BJJ practitioners suffer more injuries and are less likely to wear protective equipment during either training or competition. Future research should include larger prospective trials aimed at the use of various protective equipment for the prevention of injuries to the hand/fingers and the knee.

## References

[1] Green TA, Svinth JR (2010). Martial Arts of the World: An Encyclopedia of History and Innovation. Martial Arts of the World: An Encyclopedia of History and Innovation.

[2] Bu B, Haijun H, Yong L, Chaohui Z, Xiaoyuan Y, Singh MF (2010). Effects of martial arts on health status: a systematic review. J Evid Based Med.

[3] Petrisor BA, Del Fabbro G, Madden K, Khan M, Joslin J, Bhandari M (2019). Injury in Brazilian jiu-jitsu training. Sports Health.

[4] (2021). General System of Graduation - IBJJF. Graduation System - IBJJF -.

[5] Bledsoe GH, Hsu EB, Grabowski JG, Brill JD, Li G (2006). Incidence of injury in professional mixed martial arts competitions. J Sports Sci Med.

[6] Thomas RE, Thomas BC (2018). Systematic review of injuries in mixed martial arts. Phys Sportsmed.

[7] Kreiswirth EM, Myer GD, Rauh MJ (2014). Incidence of injury among male Brazilian jiujitsu fighters at the World Jiu-Jitsu No-Gi Championship 2009. J Athl Train.

[8] Scoggin JF 3rd, Brusovanik G, Izuka BH, Zandee van Rilland E, Geling O, Tokumura S (2014). Assessment of injuries during Brazilian jiu-jitsu competition. Orthop J Sports Med.

[9] McDonald AR, Murdock FA Jr, McDonald JA, Wolf CJ (2017). Prevalence of injuries during Brazilian jiu-jitsu training. Sports (Basel).

[10] Moriarty C, Charnoff J, Felix ER (2019). Injury rate and pattern among Brazilian jiu-jitsu practitioners: a survey study. Phys Ther Sport.

[11] Phillips J, Amosun S (2001). Injury surveillance in taekwondo and judo during physiotherapy coverage of the seventh All Africa Games. S Afr J Physiother.

[12] Ngai KM, Levy F, Hsu EB (2008). Injury trends in sanctioned mixed martial arts competition: a 5-year review from 2002 to 2007. Br J Sports Med.

[13] Stephenson C, Rossheim ME (2018). Brazilian jiu jitsu, judo, and mixed martial arts injuries presenting to United States emergency departments, 2008-2015. J Prim Prev.

[14] Ferrari CH, Ferreria de Mederios JM (2002). Dental trauma and level of information: mouthguard use in different contact sports. Dent Traumatol.

[15] Valleser CM (2016). Common injuries of recreational jiu-jitsu. J Phys Ed Res.

[16] Schweizer A (2000). Biomechanical effectiveness of taping the A2 pulley in rock climbers. J Hand Surg Br.

[17] Warme WJ, Brooks D (2000). The effect of circumferential taping on flexor tendon pulley failure in rock climbers. Am J Sports Med.

[18] Schöffl V, Schöffl I, Frank L, Küpper T, Simon M, Lutter C (2020). Tendon injuries in the hands in rock climbers: epidemiology, anatomy, biomechanics and treatment-an update. Muscles Ligaments Tendons J.

